# Association between preoperative anaemia and one year mortality risk in older patients undergoing femoral neck fracture surgery: an observational study

**DOI:** 10.1007/s00264-025-06521-4

**Published:** 2025-04-09

**Authors:** Pei-Pei Li, Ziruo Zhang, Jing Hu, Hong Zhi, Ping Xie, Xin Jiao, Dan Chen, Lian Wen

**Affiliations:** 1 Sports Medicine Diagnosis and Treatment Center, Honghui Hospital, Xi’an Jiaotong University, Xi’an City, Shaanxi Province China; 2 School of Medicine, Yan’an University, Yan’an City, Shaanxi Province China; 3 Department of Nursing, Honghui Hospital, Xi’an Jiaotong University, Xi’an City, Shaanxi Province China; 4 Emergency Department, Honghui Hospital, Xi’an Jiaotong University, Xi’an City, Shaanxi Province China; 5 Internal Medicine Ward, Honghui Hospital, Xi’an Jiaotong University, Xi’an City, Shaanxi Province China

**Keywords:** Anaemia, Haemoglobin, Femoral neck fracture, Retrospective study, Mortality risk

## Abstract

**PURPOSE:**

This research was designed to explore the incidence of anaemia before surgery and the rate of mortality one year after surgery for femoral neck fractures in older adults. It also investigated whether anaemia prior to surgery influences the likelihood of mortality within one year after the procedure.

**METHODS:**

A retrospective cohort analysis was undertaken at Honghui Hospital, a tertiary academic medical institution affiliated with Xi’an Jiaotong University in China. This investigation included elderly individuals who underwent surgery for femoral neck fractures within the year spanning from January to December 2021. The research team gathered data encompassing demographic details, levels of haemoglobin prior to surgery, existing comorbid conditions, and mortality statistics after one year.

**RESULTS:**

In this retrospective study, 994 patients were analyzed, with 84 reported fatalities. The incidence of anaemia in this group was 71.1%, affecting 707 individuals. Of these, 486 (48.8%) had mild anaemia, and 221 (22.2%) exhibited moderate to severe anaemia. Independent factors correlating with heightened one-year mortality risk included operative blood transfusions (odds ratio [OR] = 1.8, *p* = 0.0327), coronary artery disease presence (OR = 1.85, *p* = 0.0077), and moderate to severe anaemia (OR = 3.18, *p* = 0.0006). In contrast, higher body mass index (OR = 0.8, *p* < 0.0001) and red blood cell count (OR = 0.6, *p* = 0.0253) were linked to reduced one-year mortality risk. Multivariate logistic regression analyses underscored the independent association of moderate to severe anaemia with increased one-year mortality risk, with varying ORs across models: non-adjusted OR at 3.18 (*p* = 0.0006), Adjust I model OR at 3.08 (*p* = 0.0191), and Adjust II model OR at 2.96 (*p* = 0.0278).

**CONCLUSION:**

At Honghui Hospital, affiliated with Xi’an Jiaotong University in China, anemia has been identified as a common condition among elderly patients undergoing surgery for femoral neck fractures, and it significantly contributes to an elevated risk of mortality within one year post-surgery. It is advisable to implement interventions aimed at managing anaemia before surgery, which should include setting haemoglobin thresholds that are not specific to any gender for its diagnosis.

**Supplementary Information:**

The online version contains supplementary material available at 10.1007/s00264-025-06521-4.

## Introduction

Globally, hip fractures significantly contribute to morbidity and mortality, especially among those aged over 50 [1]. Such fractures are notably prevalent within the senior demographic, with femoral neck fractures accounting for 3.6% of total fracture cases [2–3]. Forecasts suggest a substantial increase in the global annual occurrence of hip fractures, potentially escalating from 1.66 million cases in 1990 to an estimated 6.26 million by 2050 [4]. Due to its anatomical position, the femoral neck is particularly vulnerable to fractures, as it forms the junction of the femoral head and the acetabulum [5]. Data reveal that femoral neck fractures constitute up to 53% of all proximal femur fractures, with a rising trend noted in their occurrence [6]. Post-fracture, the in-hospital mortality rate stands at around 6%, escalating to between 20% and 30% within the first year, peaking in the initial six months [7–8]. Moreover, the prevalence of anaemia before surgery in patients undergoing non-cardiac procedures reaches high levels, evidenced by rates of 30.4% in the U.S. and 28.7% in Europe [9]. This condition is critically concerning as even mild anaemia is associated with detrimental postoperative outcomes [9–13]. Several factors exacerbate mortality risks post-hip fracture, including comorbidities quantified by the Charlson Comorbidity Index (CCI), haemoglobin (Hgb) levels, arrhythmias, pneumonia, cardiac insufficiency, and elevated leukocyte counts [14–15]. Notably, lower haemoglobin levels upon hospital admission, as compared to normative data, are linked to increased postoperative mortality [16–18]. Yombi et al. further confirmed through adjusted analysis that a haemoglobin level below 12 g/dL at admission is independently associated with both short- and long-term postoperative mortality following hip fracture surgeries [19]. Additionally, the length of hospital stay post-fracture is a significant mortality risk for patients one year after undergoing femoral neck fracture repair, while both CCI and age remain crucial determinants of five-year postoperative mortality (*p* < 0.001 for both) [20]. Existing literature establishes a clear linkage between low preoperative Hgb concentrations and increased mortality [16, 17, 21]. Despite this, studies focusing on the relationship between preoperative anaemia and outcomes of femoral neck fracture surgeries are still limited. This investigation thus aims to delineate the impact of preoperative anaemia on the one-year mortality risk in patients who have undergone femoral neck fracture surgeries.

## Methods

### Participants and data source

From January to December 2021, a retrospective observational study was conducted at Honghui Hospital, affiliated with Xi’an Jiaotong University in China. This study was performed in line with the principles of the Declaration of Helsinki, and this study obtained the necessary ethical approval from the Ethics Committee at the Faculty of Medicine, Honghui Hospital, Xi’an Jiaotong University(No. 202403060). Patient data, encompassing clinical and biochemical profiles, were meticulously extracted from electronic health records. Eligibility for inclusion required: (1) individuals aged 60 years or older who sustained a femoral neck fracture and underwent surgical intervention, and (2) those who received any form of surgical treatment for femoral neck fractures. The study excluded cases if they: (1) involved fractures in multiple regions, (2) stemmed from high-energy trauma, (3) included patients needing revision surgery, or (4) had medical records that were incomplete or lacked sufficient data, impacting the comprehensive assessment of the case, specifically if more than 10% of the data was missing.

**Fig. 1:**
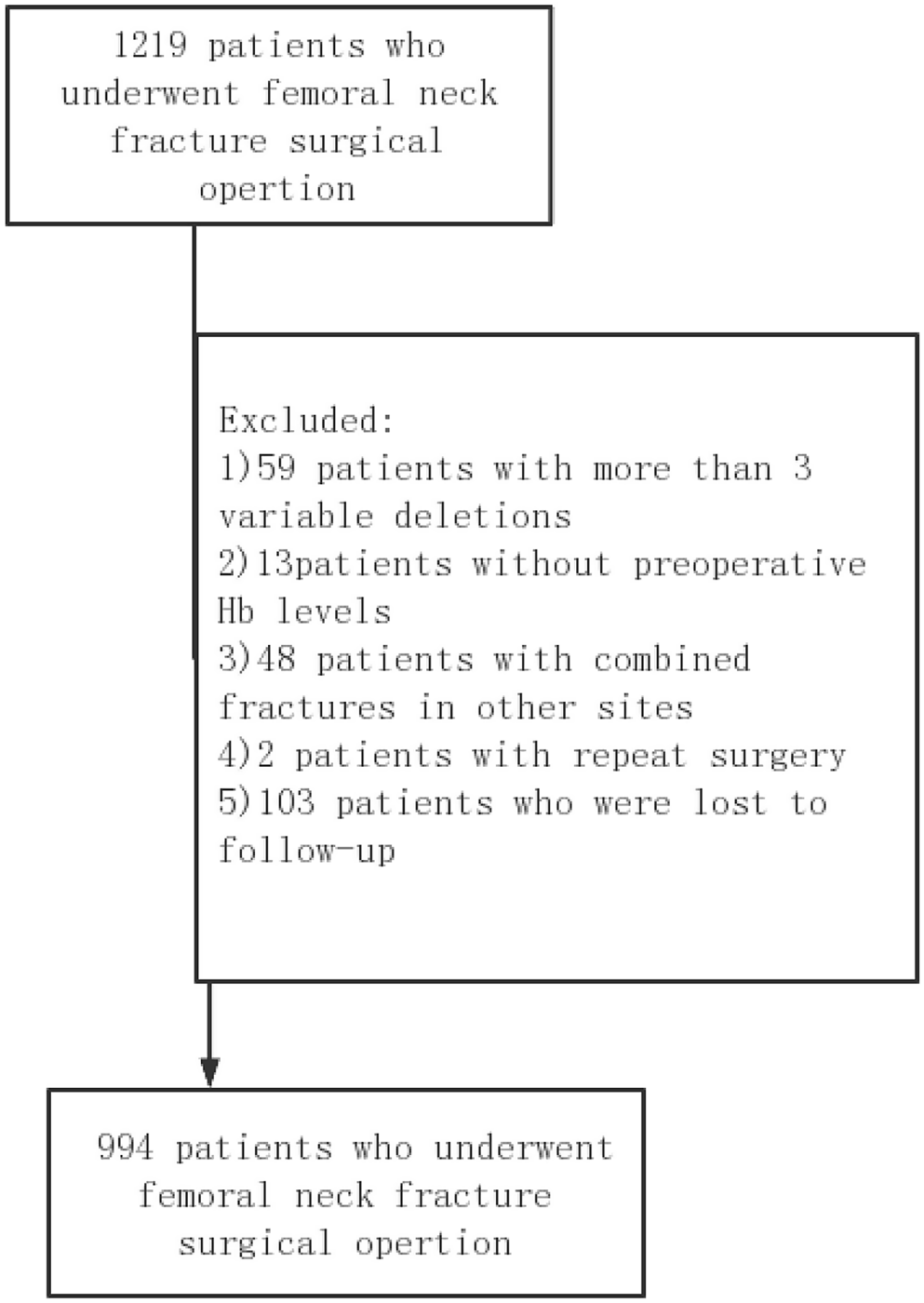
Flowchart shows the derivation of study cohort

Throughout 2021, detailed patient data were systematically gathered at Honghui Hospital, Xi’an Jiaotong University, China, for a retrospective observational study approved by the institution’s Ethics Committee(No.202403060).Informed consent was obtained from all individual participants included in the study.The collected information included demographic characteristics, existing medical conditions, the type of surgical intervention performed, anaesthesia techniques employed, details regarding intraoperative blood transfusions, scores from the American Society of Anesthesiologists (ASA), and the interval from the initial injury to the surgical procedure. For biochemical evaluations, data on haemoglobin, white blood cells, red blood cells, platelets, lymphocytes, and creatinine levels were recorded at admission, with haemoglobin concentrations specifically assessed between one and fourteen days prior to surgery. The study excluded 59 patients due to significant data omissions across more than three variables, 13 patients for the absence of preoperative haemoglobin measurements, 48 patients who presented with additional fractures at other anatomical sites, two patients who required repeat surgeries, and 103 patients who were lost to follow-up. Consequently, the final analysis encompassed data from 994 patients (refer to Fig. 1). Given the trivial extent of missing data, which amounted to only 2.0%, the necessity for a sensitivity analysis was negated. Mortality data for the one-year postoperative period were primarily extracted from hospital records, with additional verification through telephone follow-ups in instances of incomplete data. The duration from the date of the hip fracture to the occurrence of death was meticulously documented and analyzed.

### Statistical analysis

Categorical data were presented using frequencies and percentages, while continuous data were summarized through means and their corresponding standard deviations (SDs). The statistical tests applied included Fisher’s exact test for categorical variables and the Student’s t-test for those that are continuous. Univariate analyses regarding mortality rates employed a 95% confidence interval (95% CI), with statistical significance firmly established at a p-value less than 0.05. Adhering to the World Health Organization (WHO) guidelines, the seriousness of anaemia was defined based on gender-specific criteria [22]: no anemia was categorized as a haemoglobin (Hb) level of 13.0 g/dL or higher, mild anaemia was designated for Hb levels ranging from 11.0 to 12.9 g/dL, and moderate to severe anaemia was defined for Hb levels below 11.0 g/dL. The initial comparison involved the baseline clinical and biochemical characteristics of patients suffering from femoral neck fractures (Table 1). This was followed by the presentation of univariate analyses of the one-year mortality rates for these patients (Table 2). Additionally, a subgroup analysis was conducted, comparing mortality among patients with mild versus moderate to severe anemia (Table 3). A further step involved employing multivariate logistic regression to adjust for additional confounding factors when comparing mortality rates between these anaemia groups (Table 4). Analyses used R and Empower software. Ethical approval by Xi’an Honghui Hospital, approval no. 202,403,060.

## Results

### Demographics

In this study, 994 patients with femoral neck fractures qualified under the inclusion criteria. Of these, mortality was observed in 84 patients. Regarding anemia classification, 221 patients were identified with moderate to severe anaemia, 486 exhibited mild anaemia, and 287 patients were categorized as having no anaemia. As detailed in Table 1, the average age of patients who died was significantly higher, recorded at 84.5 ± 5.8 years. The length of stay for patients who died was significantly higher, recorded at 8 days. No notable differences were observed in the prevalence of hypertension, DM, CVA, dementia, or the type of anaesthesia used between the groups. Nevertheless, patients who died typically exhibited higher ASA-PS scores and longer lengths of stay (LOS) (*p* < 0.05), suggesting a more severe health condition. Additionally, variability in mortality rates was observed across different surgical types, with the highest mortality rate recorded in patients undergoing partial hip replacement and no deaths in those undergoing internal fixation (*p* < 0.001).

**Table 1:** Baseline clinical and biochemical characteristics of femoral neck fracture patients (*n* = 994)

Characteristics				Survival	Mortality	*P*-value
N				910	84	
Age: mean (SD)				74.7 ± 8.6	84.5 ± 5.8	< 0.001
Gender: n (%)			Female	651 (71.5%)	50 (59.5%)	0.021
			Male	259 (28.5%)	34 (40.5%)	
Body mass index: mean (SD)				21.5 ± 2.6	20.3 ± 2.2	< 0.001
Type of anaesthesia: n (%)			GA	903 (99.2%)	82 (97.6%)	0.300
			RA	7 (0.8%)	2 (2.4%)	
Duration of operation: minutes (SD)				95.6 ± 46.7	83.4 ± 31.9	0.019
operative blood transfusion: n(%)			None	757 (83.2%)	62 (73.8%)	0.031
			≥ one unit	153 (16.8%)	22 (26.2%)	
Surgery type: (%)		THA		288 (31.6%)	2 (2.4%)	< 0.001
		Partial-hip replacement		573 (63.0%)	82 (97.6%)	
	Internal fixation	Cannulated Screws		33 (3.6%)	0 (0.0%)	
		Intramedullary nail		7(0.8%)		
		Femoral Neck System(FNS)		8(0.9%)		
		Hip screw		1(0.1%)		
ASA score: n (%)			1	597 (65.6%)	27 (32.1%)	< 0.001
			≥ 2	313 (34.4%)	57 (67.9%)	
LOS(d), Mean ± SD				7.00 (6.00, 9.00)	8.00 (6.00, 10.00)	0.018
WBC(×10^9^/L): mean (SD)				7.49 ± 2.47	7.57 ± 2.45	0.783
RBC(×10^12^/L): mean (SD)				4.0 ± 0.6	3.8 ± 0.7	0.027
LYMPH (×10^9^/L): mean (SD)				1.2 ± 0.6	1.1 ± 0.5	0.136
creatinine (umol/L): mean (SD)				63.2 ± 26.5	72.3 ± 29.7	0.003
Anaemia: n (%)			None	273 (30.0%)	14 (16.7%)	0.001
			Mild	447 (49.1%)	39 (46.4%)	
			Moderate/severe	190 (20.9%)	31 (36.9%)	
Hypertension: n (%)			NO	498 (54.7%)	40 (47.6%)	0.431
			YES	412 (45.3%)	44 (52.4%)	
Coronary heart disease: n (%)			NO	629 (69.1%)	46 (54.8%)	0.007
			YES	281 (30.9%)	38 (45.2%)	
DM: n (%)			NO	732 (80.4%)	64 (76.2%)	0.351
			YES	178 (19.6%)	20 (23.8%)	
CVA: n (%)			NO	672 (73.8%)	61 (72.6%)	0.807
			YES	238 (26.2%)	23 (27.4%)	
Dementia: n (%)			NO	881 (96.8%)	78 (92.9%)	0.060
			YES	29 (3.2%)	6 (7.1%)	

(SD, standard deviation; GA, general anaesthesia; RA, regional anaesthesia; THA, Total Hip Arthroplasty; WBC, white blood cell: RBC, red blood cell: LYMPH, lymphocyte;ASA score, American Society of Anesthesiologists (ASA) Physical Status Classification: LOS, Length of stay; DM, diabetes mellitus; CVA, cerebrovascular accidents.)

### Effect size of haemaglobin on one year mortality

**Fig. 2:**
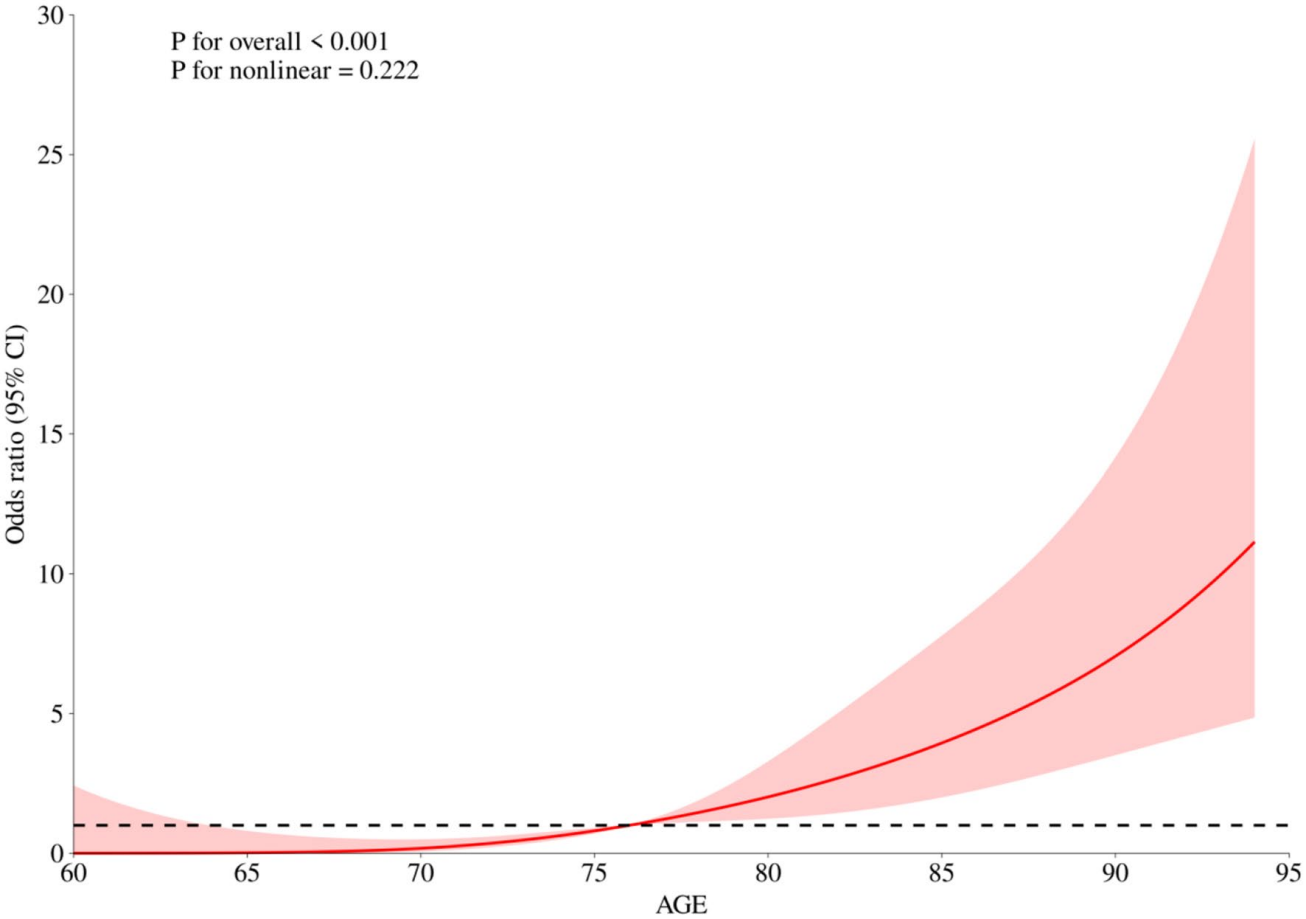
The RCS curve for age and mortality

Multiple independent determinants of one-year mortality were identified in this study. Notably, patients who received surgical blood transfusions were more likely to experience higher mortality, with an odds ratio of 1.8 (*p* = 0.0327). Additionally, the risk of death increased with age, with an odds ratio of 1.2 (*p* < 0.0001). Restricted Cubic Splines (RCS) were generated using R version 4.4.0 in this study. Our analysis identified 76 years as a critical age threshold, beyond which the risk of death significantly increased, as shown in Fig. 2.Furthermore, those diagnosed with coronary heart disease had an increased risk, OR = 1.85 (*p* = 0.0077). Moderate to severe anemia was also a significant predictor, showing an OR of 3.18 (*p* = 0.0006). Conversely, an elevated body mass index was associated with a lower one-year mortality risk (OR = 0.8, *p* < 0.0001), as was a higher red blood cell count, which had an OR of 0.6 (*p* = 0.0253). The analysis of mild anemia and hypertension did not reveal a statistically significant relationship with one-year mortality rates, as presented in Table 2.

Table 3 illustrates the breakdown of risk factors significantly increasing the one-year mortality risk among various patient subgroups. For females with moderate to severe anaemia, the OR was 2.68 (*p* = 0.0427), while for males it was markedly higher at 6.55 (*p* = 0.0001). Patients with hypertension and moderate to severe anaemia had an OR of 3.49 (*p* = 0.0092), and those with coronary heart disease paired with moderate to severe anaemia had an even higher OR of 4.00 (*p* = 0.0095). Moreover, patients with an ASA score of 1 and moderate to severe anaemia had an OR of 3.52 (*p* = 0.0437), and for those receiving one or more units of blood during surgery, the risk escalated dramatically to an OR of 17.97 (*p* = 0.0065).

Further analysis through multivariate logistic regression confirmed that moderate to severe anaemia was a potent independent predictor of increased one-year mortality risk. The non-adjusted model indicated an OR of 3.18 (*p* = 0.0006), and the adjusted models I and II showed ORs of 3.08 (*p* = 0.0191) and 2.96 (*p* = 0.0278), respectively, as shown in Table 4.

**Table 2:** Univariable analysis of 1-Year mortality in femoral neck fracture patients

Variable		OR (95%CI)	*P*-value
Age		1.2 (1.1, 1.2)	< 0.0001
Gender	Female	REF	REF
	Male	1.7 (1.1, 2.7)	0.0220
Body mass index		0.8 (0.7, 0.9)	< 0.0001
Type of anaesthesia	GA	REF	REF
	RA	3.15 (0.64, 15.39)	0.1571
Duration of operation		1.0 (1.0, 1.0)	0.0123
Operative blood transfusion	None	REF	REF
	≥ one unit	1.8 (1.0, 2.9)	0.0327
Surgery type	THA	REF	REF
	Partial-hip replacement	20.6 (5.0, 84.4)	< 0.0001
	Internal fixation	0.0 (0.0, 0.0)	0.9822
ASA score	1	REF	REF
	≥ 2	4.03 (2.50, 6.49)	< 0.0001
WBC(×10^9^/L)		1.01 (0.93, 1.11)	0.7824
RBC(×10^12^/L)		0.6 (0.4, 0.9)	0.0253
LYMPH (×10^9^/L)		0.68 (0.42, 1.10)	0.1167
creatinine (umol/L)		1.01 (1.00, 1.01)	0.0177
LOS (d)		1.01(0.98,1.04)	0.4850
Anaemia	None	REF	REF
	Mild	1.70 (0.91, 3.19)	0.0977
	Moderate/severe	3.18 (1.65, 6.14)	0.0006
Hypertension	NO	REF	REF
	YES	1.33 (0.85, 2.08)	0.2123
Coronary heart disease	NO	REF	REF
	YES	1.85 (1.18, 2.91)	0.0077
DM	NO	REF	REF
	YES	1.29 (0.76, 2.18)	0.3519
CVA	NO	REF	REF
	YES	1.06 (0.64, 1.76)	0.8068
Dementia	NO	REF	REF
	YES	2.34 (0.94, 5.80)	0.0672

**Table 3:** Univariable analysis of mortality comparing mild anaemia and moderate/severe anaemia (Subgroup analysis)

Sub-group			Nurber	Mortality	
				OR (95%CI)	*P*-value
Gender	Female	None	146	REF	REF
		Mild	380	1.71 (0.69, 4.25)	0.2454
		Moderate/severe	175	2.68 (1.03, 6.93)	0.0427
	Male	None	141	REF	REF
		Mild	106	2.32 (0.93, 5.83)	0.0723
		Moderate/severe	46	6.55 (2.51,17.10)	0.0001
Hypertension	YES	None	134	REF	REF
		Mild	228	1.94 (0.80, 4.67)	0.1401
		Moderate/severe	93	3.49 (1.36, 8.94)	0.0092
Coronary heart disease	YES	None	75	REF	REF
		Mild	163	1.42 (0.50, 4.06)	0.5142
		Moderate/severe	81	4.00 (1.40, 11.40)	0.0095
DM	YES	None	63	REF	REF
		Mild	101	2.70 (0.73, 9.96)	0.1368
		Moderate/severe	34	3.45 (0.77, 15.43)	0.1054
CVA	YES	None	85	REF	REF
		Mild	121	0.93 (0.31, 2.79)	0.9001
		Moderate/severe	55	2.58 (0.86, 7.70)	0.0903
Dementia	YES	None	9	REF	REF
		Mild	15	2.00 (0.18, 22.80)	0.5767
		Moderate/severe	11	1.78 (0.13, 23.52)	0.6623
ASA score	1	None	201	REF	REF
		Mild	303	2.57 (0.84, 7.84)	0.0986
		Moderate/severe	120	3.52 (1.04, 11.94)	0.0437
	≥ 2	None	86	REF	REF
		Mild	183	1.15 (0.52, 2.52)	0.7323
		Moderate/severe	101	2.24 (1.00, 5.02)	0.0500
operative blood transfusion	None	None	239	REF	REF
		Mild	406	1.44 (0.74, 2.80)	0.2875
		Moderate/severe	174	2.01 (0.96, 4.21)	0.0659
	≥ one unit	None	48	REF	REF
		Mild	80	5.22 (0.63, 43.12)	0.1249
		Moderate/severe	47	17.97 (2.24, 144.02)	0.0065

**Table 4:** Multivariate logistic regression of mortality comparing mild anaemia and moderate/severe anaemia and adjusting for factors

anaemia categorical	Non-adjusted		Adjust I		Adjust II	
	OR (95%CI)	*P*-value	OR (95%CI)	*P*-value	OR (95%CI)	*P*-value
None	REF	REF	REF	REF	REF	REF
Mild	1.70(0.91, 3.19)	0.0977	1.56 (0.72, 3.37)	0.2618	1.63 (0.74, 3.57)	0.2244
Moderate/severe	3.18(1.65, 6.14)	0.0006	3.08 (1.20, 7.91)	0.0191	2.96 (1.13, 7.78)	0.0278

Crudel model: None of the variables were adjusted; Adjust I adjust for: Age; Gender; Adjust II adjust for: Age; Gender; Hypertension; Coronary heart disease; Diabetes mellitus; Cerebrovascular accidents; Dementia; ASA score; Type of anaesthesia; Surgery type; Duration of operation; White blood cell; Red blood cell; creatinine; BMI; Operative blood transfusion

## Discussion

A detailed retrospective cohort analysis was conducted at our medical centre, involving 994 consecutive patients who underwent elective unilateral primary fixation for femoral neck fractures (FNF). The study revealed a one-year mortality rate of 8.45%, notably lower than the previously reported range of 15–50% for similar procedures [23–24]. In contrast, the postoperative mortality rate for internal fixation patients in our study was 0%. This difference may be attributed to the fact that our study population was composed of individuals aged over 60 years, with exclusion criteria that eliminated high-energy trauma cases and revision surgeries. Additionally, the standardized perioperative protocol at our institution may have contributed to the lower mortality rate observed. We acknowledge the potential for selection bias, which represents a limitation of the study, and recommend that further multicenter studies be conducted to validate these findings. Furthermore, internal fixation patients were younger (mean age: 65.24 ± 5.43 vs. 79.33 ± 7.46 for hemi-hip replacement) and had lower ASA scores (89.58% vs. 50.54% for hemi-hip arthroplasty) (as shown in Attached Table 1). These factors may have contributed to the more favourable postoperative recovery. The variability in mortality rates observed in previous studies emphasizes the challenges of drawing definitive conclusions without comprehensive data stratification and analysis. The presence of multiple confounding risk factors further complicates the evaluation, necessitating a more detailed approach to assess the impact of each identified risk factor on patient outcomes.

Effective perioperative management is crucial in determining outcomes such as postoperative complications and mortality rates. Nevertheless, a significant number of research studies focused on mortality following femoral neck fractures have neglected to thoroughly explore the nuances of perioperative strategies and their subsequent effects. Our research highlights that moderate to severe anaemia in male patients significantly forecasts increased postoperative mortality. Particularly, this condition correlates strongly with other severe health issues like hypertension (odds ratio [OR] = 3.49, *p* = 0.0092), coronary heart disease (OR = 4.00, *p* = 0.0095), an ASA score of 1 (OR = 3.52, *p* = 0.0437), and the necessity for multiple units of blood during surgery (OR = 17.97, *p* = 0.0065). This aligns with findings from Kastanis et al., which identified ASA status as a prognostic marker for medical complications and hospital readmissions in geriatric patients with hip fractures [26]. Although our study employed a stratified analysis to delve deeper into these associations, it is noteworthy that such detailed stratifications are often under reported in the literature.

In the observed study, 221 patients exhibited moderate to severe anemia, while 486 presented with mild anaemia, and 287 displayed no signs of anaemia. Research conducted by Zhang Hui and colleagues [27] identified the prevalence of preoperative anaemia among patients suffering from femoral and pelvic fractures as 47.8%. However, in our analysis, the prevalence rate escalated to 75.2% among individuals with femoral shaft fractures, indicating a higher occurrence rate of preoperative anaemia at this specific anatomical site.The impact of anaemia on the likelihood of mortality within one year following femoral neck fracture surgeries in the elderly population showed variability. Specifically, among elderly patients suffering from hip fractures, those identified with anaemia—defined as haemoglobin concentrations below 12.0 g per deciliter for female patients and below 13.0 g per deciliter for male patients—demonstrated a higher probability of being nonambulatory at the time of hospital discharge when contrasted with their non-anaemic counterparts [28]. Contrarily, another study noted no significant variance in the recovery levels regarding ambulation or daily living activities at three, six, or twelve months post-surgery between anaemic and non-anaemic subjects [29]. An earlier study highlighted that while admission haemoglobin levels correlated with mobility on a univariate scale, this correlation faded under multivariate analysis [30]. The lack of significant outcomes across these studies could stem from the differing anaemia severity levels among participants. It is revealed through our investigation that only moderate to severe anaemia has a consistent impact on the risk of mortality within a year, while the influence of mild anaemia appears more subdued. Neglecting to identify and address preoperative anaemia promptly can lead to a surge in blood transfusions, an increased risk of infections, higher residual rates, and elevated mortality rates, alongside prolonged hospitalization durations and diminished postoperative functionality. Even mild anaemia can significantly heighten the risk of developing these complications [31–32].

Our investigation established that for patients experiencing moderate to severe anaemia, a rise of 1 g/dL in preoperative haemoglobin (Hb) levels is linked to an approximately threefold (2.96 times) increase in the likelihood of mortality within the subsequent year. This strong association persists robustly after controlling for confounding factors such as age, gender, blood pressure, and pre-existing coronary conditions, highlighting preoperative anaemia as a potent independent predictor of mortality risk. Further investigations corroborate these findings, showing that mild anaemia at the time of admission is linked to a 50% increase in mortality within three months (95% CI: 1.1–1.9). The risk escalates with the severity of anaemia; moderate anaemia correlates with a 2.6 times higher risk (95% CI: 2.0–3.4), and severe anaemia corresponds to a 3.6 times increase (95% CI: 1.8–6.9) in mortality compared to individuals without anaemia [33]. These data are particularly relevant to healthcare systems in China, where demographic patterns, such as an aging population, can influence outcomes. Our study also found that age is a risk factor for postoperative death in elderly femoral neck fracture patients, with the risk increasing with age. A critical age threshold of 76 years was identified, with a significantly increased risk of death after this age, as confirmed in other studies of Bzovsky et al. [34]. This increased in risk may be due to the decline in bodily functions, weakening of immune tolerance, and reduced self-healing abilities, leading to an increased risk of complications and eventually increased mortality. Haemoglobin levels prove to be a vital, cost-effective metric for predicting mortality among elderly patients who suffer from femoral neck fractures. Leveraging this prognostic factor could substantially improve healthcare outcomes. However, it is pertinent to note that our study did not delve into analyzing the specific causes of death, which could provide further insights into the correlations observed.

## Conclusion

Data indicated that lower haemoglobin concentrations upon hospital admission were linked to elevated mortality rates one year following admission. In particular, cases classified as moderate to severe anaemia demonstrated a nearly threefold heightened mortality risk, with a 95% confidence interval ranging from 1.13 to 7.78. Therefore, moderate to severe anaemia could be recognized as an effective and economical indicator for forecasting mortality among elderly individuals undergoing surgery for femoral neck fractures. It is recommended that subsequent studies explore the consequences of moderate to severe anaemia on mortality rates post-surgery in such patients.

### Limitations

This study has several constraints that should be noted: (1) The analysis was carried out as a retrospective review of data collected at a solitary level I Trauma Center. This aspect may restrict the extent to which our findings can be extrapolated to broader contexts. Moreover, the cohort primarily comprised Chinese individuals, which might limit how applicable the results are to diverse international populations. (2) Many of the follow-up engagements were conducted through telephone communications. This method likely contributed to attrition in our follow-up data, as some relatives of deceased patients were reticent to communicate, leading to potential underestimations of mortality rates and inaccuracies in assessing mortality-related risk factors.(3) Gaps in the recording of femoral neck fracture subtypes in this study precluded a meaningful analysis of death risk stratified by Garden classification. Future prospective studies should prioritize standardized documentation of fracture subtypes to further elucidate their prognostic significance.

## Electronic supplementary material

Below is the link to the electronic supplementary material.

Supplementary Material 1

## Data Availability

No datasets were generated or analysed during the current study.
