# Supplementary material for: Association between preoperative anaemia and one year mortality risk in older patients undergoing femoral neck fracture surgery: an observational study
Source: Int Orthop. 2025 Apr 9;49(6):1483–91. doi: 10.1007/s00264-025-06521-4 (PMC12075266; doi:10.1007/s00264-025-06521-4)
Supplement: Supplementary file 1 — Supplementary Material 1 [file 264_2025_6521_MOESM1_ESM.docx]

**Attached table 1 .**Comparison of relevant data on operation types in elderly patients with femoral neck fracture

| Variables | THA (n = 290) | Partial-hip replacement (n = 655) | Internal fixation (n = 49) | *P* |
| --- | --- | --- | --- | --- |
|  |  |  |  |  |
| AGE, Mean ± SD | 68.54 ± 5.91 | 79.33 ± 7.46 | 65.24 ± 5.43 | **< .001** |
| ASA score :n (%) |  |  |  | **< .001** |
| 1 | 247(85.02) | 333(50.54) | 44 (89.58) |  |
| ≥ 2 | 43 (14.98) | 322 (49.46) | 5 (10.42) |  |
